# Human tissue-resident peritoneal macrophages reveal resistance towards oxidative cell stress induced by non-invasive physical plasma

**DOI:** 10.3389/fimmu.2024.1357340

**Published:** 2024-03-05

**Authors:** Laura Schultze-Rhonhof, Julia Marzi, Daniel Alejandro Carvajal Berrio, Myriam Holl, Theresa Braun, Felix Schäfer-Ruoff, Jürgen Andress, Cornelia Bachmann, Markus Templin, Sara Y. Brucker, Katja Schenke-Layland, Martin Weiss

**Affiliations:** ^1^Department of Women’s Health Tübingen, University of Tübingen, Tübingen, Germany; ^2^Institute of Biomedical Engineering, Department for Medical Technologies and Regenerative Medicine, University of Tübingen, Tübingen, Germany; ^3^Natural and Medical Sciences Institute (NMI) at the University of Tübingen, Reutlingen, Germany; ^4^University Development, Research and Transfer, University of Konstanz, Konstanz, Germany

**Keywords:** non-invasive physical plasma (NIPP), cold atmospheric plasma (CAP), plasma-activated media (PAM), plasma-treated solutions (PTS), human primary macrophages, immune response, peritoneal cavity, peritoneal cancer

## Abstract

In the context of multimodal treatments for abdominal cancer, including procedures such as cytoreductive surgery and intraperitoneal chemotherapy, recurrence rates remain high, and long-term survival benefits are uncertain due to post-operative complications. Notably, treatment-limiting side effects often arise from an uncontrolled activation of the immune system, particularly peritoneally localized macrophages, leading to massive cytokine secretion and phenotype changes. Exploring alternatives, an increasing number of studies investigated the potential of plasma-activated liquids (PAL) for adjuvant peritoneal cancer treatment, aiming to mitigate side effects, preserve healthy tissue, and reduce cytotoxicity towards non-cancer cells. To assess the non-toxicity of PAL, we isolated primary human macrophages from the peritoneum and subjected them to PAL exposure. Employing an extensive methodological spectrum, including flow cytometry, Raman microspectroscopy, and DigiWest protein analysis, we observed a pronounced resistance of macrophages towards PAL. This resistance was characterized by an upregulation of proliferation and anti-oxidative pathways, countering PAL-derived oxidative stress-induced cell death. The observed cellular effects of PAL treatment on human tissue-resident peritoneal macrophages unveil a potential avenue for PAL-derived immunomodulatory effects within the human peritoneal cavity. Our findings contribute to understanding the intricate interplay between PAL and macrophages, shedding light on the promising prospects for PAL in the adjuvant treatment of peritoneal cancer.

## 1 Introduction

Non-invasive physical plasma (NIPP), a highly reactive gas at near room temperature, can be applied directly to solids (direct treatment) or transferred from gas to liquid phase (indirect treatment) to propagate plasma-activated liquids (PAL) (1, 2). Biologically active reagents (e.g., reactive oxygen and nitrogen species, RONS) are formed at the interface of plasma discharge, surrounding air and the target (3), inducing dose-dependent anti-proliferative, selective anti-tumoral and wound healing or regenerative effects at a cellular and tissue level (4–7).

Research on human tissue-resident macrophages is scarce due to the increased difficulty of isolation and culture (e.g., surgical procedures, low cell counts) (8). Findings, therefore, largely originate from *in vitro* monocyte-derived or murine macrophages (9), of which fate-mapping studies revealed that in a homeostatic state, the population of tissue-resident macrophages primarily comprises large peritoneal macrophages (LPMs) (10). One-tenth of the population consists of small blood monocyte-derived peritoneal macrophages (SPMs). Differently from SPMs, LPMs stem from yolk-sac progenitors and have self-renewal potential with GATA-binding protein 6 (GATA-6), a transcription factor, responsible for their differentiation and survival (11). Owing to their high plasticity, tissue-resident macrophages can initiate an immune response, regulate wound repair and modulate tumor expansion (12). “Classically” activated (M1) macrophages exert cytotoxic effects, express CD86, a co-stimulatory molecule required for the activation of T cells, and release pro-inflammatory cytokines (e.g., IL-6, IL-17) (13–15). “Alternatively” activated (M2) macrophages can be phenotypically characterized by the scavenger receptor CD163 and have pro-tumoral properties (15–17). The M1/M2 model, however, largely applies to the *in vitro* culture of monocyte-derived macrophages activated with specific factors, whereas *in vivo* macrophages may express a larger spectrum of phenotypes with overlapping properties (12, 18). Polarization of murine macrophages towards an M1-like phenotype demonstrated cytotoxic effects and slowed tumor progression in peritoneal tumor models (19), whereas M2-like macrophages were shown to promote tumor dissemination in gastric cancer via EGFR signaling pathways (20).

Peritoneal macrophages are thus a promising target for PAL-derived immunomodulatory effects. Further research is required for the clinical use of PAL within the human peritoneal cavity for the treatment of cancerous and non-cancerous lesions including inflammatory diseases.

## 2 Materials and methods

### 2.1 Isolation and culture of human peritoneal macrophages

Peritoneal lavages were obtained after written informed consent from patients undergoing surgical procedures at the University Women’s Hospital in Tübingen. The use of human donor cells was approved by the ethics committee of the medical faculty at the Eberhard Karl’s University Tübingen (495/2018BO2). Cells were isolated from these peritoneal lavages as previously reported by Ruiz-Alcaraz et al. (21). 2 - 4 x 10^5^ cells were then seeded onto 48- well plates and left to adhere for 2 h at 37 °C and 5% CO_2_. Non-adherent cells were aspirated and removed. The plastic-adherent macrophages were washed with warm DPBS and cultured in DMEM Glutamax™ supplemented with 100 μg/mL streptomycin, 100 U/mL penicillin, 20 ng/mL macrophage-colony stimulating factor (M-CSF), 2 mM L-glutamine and 10% heat-inactivated FBS (all from ThermoFisher Scientific, OR, USA).

### 2.2 Generation of PAL and cell treatment

2 mL of Minimal Essential Medium (MEM) without pyruvate (ThermoFisher Scientific, OR, USA, #31095029) supplemented with 100 μg/mL streptomycin, 100 U/mL penicillin, 2 mM L-glutamine and 10% heat-inactivated FBS was activated by plasma exposure using an ambient pressure argon plasma jet (kINPen MED, neoplas med, Germany) for 120 s. Following operating conditions were applied: argon gas flow 4.0 L/min, frequency 1 MHz, line voltage 2-3 kV, power 1 W. 2 mL MEM were treated with pure argon gas and used as a control. An argon-treated control, 1:2-diluted and undiluted PAL were performed for experiments (excluding immunostaining and flow cytometric characterization of macrophages). In a 48- well plate, cells were incubated with 200 µL PAL for 4 h at 37 °C and 5% CO_2_ before further propagation in culture media for 24 h in total.

### 2.3 Immunofluorescence microscopy

Macrophages were harvested with Accutase (BioLegend, San Diego, CA, USA, #423201) and reseeded in glass bottom imaging dishes (μ-dish 35 mm, high glass bottom, ibidi, Germany, #81158). Cells were cultured for 24 h prior to fixation with 4% PFA for 10 min. Cells were washed three times with cold DPBS and permeabilized with ice-cold 100% methanol for 20 min at -20 °C. Cells were rinsed with cold DPBS for 5 min and blocked with a blocking buffer (0.5 g BSA + 30 μL Triton + 10 mL DPBS) for 60 min at room temperature (RT) in dark. After the blocking buffer was removed, cells were incubated overnight at 4 °C with a primary antibody diluted in antibody dilution buffer (0.1 g BSA + 30 μL Triton + 10 mL DPBS). The following primary antibody was used: Rabbit (Rb) CD68 (clone D4B9C-specific antibody, Cell Signaling Technology, Netherlands, #76437, 1:800 dilution). Cells were washed three times with DPBS and incubated with diluted secondary antibody for 60 min at RT in dark. The following fluorochrome-conjugated secondary antibody was used: Cy™3 AffiniPure Goat Anti-Rabbit IgG (H + L) (Jackson ImmunoResearch, UK, #111-165-003, 1:500 dilution). Cells were washed three times with DPBS and were incubated with the diluted nuclei-specific dye Hoechst 34580 (ThermoFisher Scientific, OR, USA, #H21486, dilution 1:1000) for 20 min on a plate shaker covered in aluminum foil. Cells were washed with DPBS prior to image acquisition with a Cell Observer fluorescent microscope (Zeiss, Germany).

### 2.4 Flow cytometric characterization

Macrophages were harvested with Accutase, washed and resuspended in 500 μL DPBS containing 0.5 μL Zombie NIR, a fixable viability dye, for 20 min at RT in dark. After washing cells twice with FACS buffer (DPBS + 2% FBS + 0.05 mM NaN_3 + _0.1 mM EDTA), cells were resuspended in 50 μL of surface marker antibodies diluted at a 1:50 dilution ratio in FACS buffer supplemented with 10% sterile-filtered, human male AB serum (H2B, France, #21001PM) for 30 min on ice in dark. The following fluorochrome-conjugated antibodies targeted against surface markers were used: CD14-PE (clone HCD14-specific antibody, BioLegend, CA, USA, #325605, dilution 1:50), CD14-FITC (clone HCD14-specific antibody, BioLegend, CA, USA, #325603, dilution 1:50) and CD16-BV605™ (clone 3G8-specific antibody, BioLegend, CA, USA, #302039, dilution 1:50). After washing, cells were resuspended in 100 μL Cytofix/Cytoperm (Fixation/Permeabilization Solution Kit, BD Bioscience, Germany, #554714). Cells were then washed twice with 1 mL 1x Perm/Wash and incubated with 100 μL blocking reagent (10% human male AB serum in 1x Perm/Wash) for 20 min on ice in dark. Intracellular antibodies were added directly to the blocking reagent. Cells were incubated with intracellular antibodies for 30 min on ice in dark. The following fluorochrome-conjugated antibodies targeted against intracellular markers were used: GATA-6-PE (clone D61E4-specific antibody, Cell Signaling Technology, Netherlands, #26452, dilution 1:50) and CD68-PE-eFluor 610 (clone Y1/82A-specific antibody, ThermoFisher Scientific, OR, USA, #61-0689-42, dilution 1:50). Cells were washed once with 1x Perm/Wash and resuspended in 100 μL FACS buffer for data acquisition using LSRFortessa™ Cell Analyzer (BD Biosciences, NJ, USA). Single-color compensation controls were performed with UltraComp eBeads™ (ThermoFisher Scientific, OR, USA, #01-3333-41) for software-based automatic compensation and adjustment of PMT voltages. Data was analyzed with FlowJo™ 10.4.2 software (Tree Star, OR, USA). Gating strategy included the removal of cell debris (FSC vs SSC), doublets (FSC-A vs SSC-A) and dead cells (FSC vs Zombie NIR) to determine positive cell populations (**Supplementary Figure S1**). FC staining of only surface markers is reported below (section 3.8).

### 2.5 Raman microspectroscopic analysis

Macrophages were harvested with Accutase and reseeded in glass bottom imaging dishes. 24 h after PAL treatment cells were fixed with 4% PFA for 10 min. Raman imaging was performed using a customized inverted WITec Raman system (alpha 300 R, WiTec GmbH, Ulm, Germany) equipped with a green laser (532 nm) and a charged-coupled device spectrograph with a grating of 600 g/mm. Large area scans were acquired of 9-10 single cells for each argon-treated control, 1:2-diluted and undiluted PAL-treated macrophages with a 63 x apochromat water dipping objective (N.A. 1.4; Olympus, Japan), an integration time of 0.1 s, a pixel resolution of 1 x 1 μm and a laser power of 50 mW. Image analysis was performed with the Project FIVE 5.1 software (WITEC GmbH, Germany), including baseline correction, removal of cosmic rays and cropping of spectra from 300 to 3045 cm^-1^. True component analysis (TCA) identified prominent spectral components, of which single spectra were extracted using TCA-generated masks from intensity distribution heat maps. Principal component analysis (PCA) was performed as previously reported with the Unscrambler x 14.0 software (Camo Software, AS, Norway) to improve interpretability of the spectral data (22, 23).

### 2.6 Apoptosis; Apotracker/7-AAD co-staining

Macrophages were harvested with Accutase 24 h after PAL treatment, washed and incubated with 400 nM Apotracker staining solution (BioLegend, CA, USA, #427401) diluted in 100 μL FACS buffer for 20 min at RT in dark. After washing cells twice with FACS buffer, cells were resuspended in 100 μL FACS buffer. Cells were stained with 5 μL of 7-AAD viability dye (BioLegend, CA, USA, #420403) for 10 min at RT in dark, which was added directly to the cell suspension prior to data acquisition with LSRFortessa™ Cell Analyzer. Data was analyzed using FlowJo™ 10.4.2 software. Apotracker/7-AAD co-staining allowed for the discrimination of early and late apoptotic, necrotic and viable cells as a percentage of total cells.

### 2.7 Protein expression analysis by DigiWest multiplex protein profiling

Macrophages were harvested with Accutase 24 h after PAL treatment. Cell pellets were frozen at -80 °C prior to DigiWest multiplex protein profiling. The high-throughput bead-based Western blot was performed as previously reported by Ruoff et al. (24). Antibody fluorescence intensities were analyzed with the Luminex™ FlexMAP 3D™ Instrument System (Luminex Corporation, TX, USA). An Excel macro-based algorithm identified peaks at the respective molecular weight of the primary antibodies. Streptavidin conjugates were recorded as loading controls to normalize antibody signals.

### 2.8 FC surface marker expression analysis

Macrophages were harvested with Accutase 24 h after PAL treatment, washed and stained. Following fluorochrome-conjugated specific antibodies targeted against surface markers were used: CD86-PE (clone IT2.2-specific antibody, BioLegend, CA, USA, #305405), HLADR-FITC (clone Tü36-specific antibody, BioLegend, CA, USA, #361603), CD206-BV421™ (clone 15-2-specific antibody, BioLegend, CA, USA, #321125) and CD163-PE/Cy7 (clone GHI/61-specific antibody, ThermoFisher, OR, USA, #25-1639-42). Antibodies were diluted with FACS buffer supplemented with 10% human male AB serum at a 1:50 dilution ratio for 30 min on ice in dark. After washing, cells were resuspended in 100 μL FACS buffer. 1 μL 7-AAD viability dye was added. Cells were incubated with 7-AAD for 10 min on ice in dark prior to FC analysis. In addition to single-color compensation controls, FMO (fluorescence minus one) controls were performed. Gating strategy included the removal of cell debris (FSC vs SSC), doublets (FSC-A vs SSC-A) and dead cells (FSC vs 7-AAD) to determine MFIs.

### 2.9 Cell culture supernatant analysis

Cell culture supernatants were collected 24 h after PAL treatment, centrifuged at 3000 x g for 3 min and stored at -80 °C until analysis. Levels of 13 different cytokines and chemokines were determined using the LEGENDplex™ HU Essential Immune Response Panel (BioLegend, San Diego, USA, #740930). The bead-based immunoassay was performed as reported in the manufacturer’s instructions. MFIs and absolute concentrations of the cytokines/chemokines were measured as technical replicates (duplicates) using LSRFortessa™ Cell Analyzer and analyzed with the LEGENDplex™ data analysis software.

### 2.10 Statistical analysis

Statistical comparison was performed with the Student’s *t*-test or Mann-Whitney *U* test against the argon-control group (GraphPad Prism 9.2.0. GraphPad Software Inc., San Diego, CA, USA). The data is shown as mean ± standard deviation of a minimum of three independent experimental approaches. *P*-values of < 0.05 were referred to as statistically significant.

## 3 Results

### 3.1 Human tissue-resident peritoneal macrophages reveal a heterogenous cellular morphology and co-expression of pro- and anti-inflammatory surface markers

**Figure 1:**
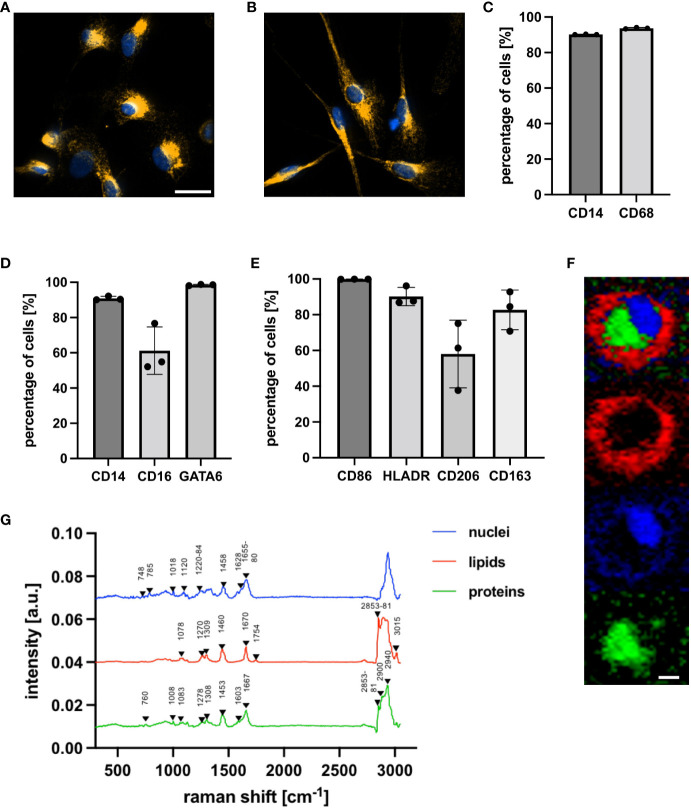
Characterization of human tissue-resident peritoneal macrophages with IF microscopy, FC staining and Raman microspectroscopy. **(A, B)** Representative IF microscopy (63 x) after staining with specific antibodies against CD68 (orange) and Hoechst, a nuclear-specific dye (blue), five days after isolation. Macrophages show round shapes and spindle-like elongation. Scale bar represents 20 µm. **(C–E)** FC analysis was used to characterize surface and intracellular markers of peritoneal macrophages. **(C, D)** shows the percentage of cells positive for the surface markers CD14 and CD16 and the intracellular pan-macrophage marker CD68 **(C)** and peritoneal macrophage-specific marker GATA-6 **(D)**. **(E)** shows the percentage of cells positive for M1 (CD86, HLADR) and M2 (CD206, CD163) surface markers. Statistical comparison was performed with paired Student’s *t*-tests. Shown are the mean ± SD, n = 3. **(F, G)** Raman microspectroscopic analysis was used to characterize peritoneal macrophages at a nuclei, lipid and protein level. **(F)** True component analysis (TCA) based on specific Raman peaks facilitated identification of nuclei (blue), lipids (red) and proteins (green) by producing false color-coded intensity distribution maps. Scale bar represents 7 µm. **(G)** Average spectra of cellular structures.

Human peritoneal macrophages were characterized with IF microscopy, FC staining and Raman microspectroscopy. IF microscopy with the intracellular, pan-macrophage marker CD68 demonstrated a heterogenous cellular morphology of the isolated peritoneal macrophages (**Figures 1A, B**). These became increasingly adherent after isolation, adopting either a round shape or a spindle-shaped elongation. FC staining with CD68 demonstrated that peritoneal macrophages represented the largest population of isolated cells (**Figure 1C**), of which co-staining with CD14, CD16 and GATA-6 showed that the majority had a high expression of CD14 and CD16 (**Figure 1D**). GATA-6 was highly expressed, indicating that primarily tissue-resident LPMs were isolated. Simultaneous FC staining of the following surface markers, CD86, HLA-DR (M1), CD206 and CD163 (M2), showed that peritoneal macrophages co-express pro- and anti-inflammatory surface markers in a homeostatic environment (**Figure 1E**). Peritoneal macrophages showed a higher basal expression of M1 surface markers. 99.9 ± 0.1% of the peritoneal macrophages expressed CD86, while 90.2 ± 5.1% of the cells were positive for HLADR. The basal expression of M2 surface markers was lower with 58.1 ± 18.9% of the cells expressing CD206 and 82.7 ± 11.1% expressing CD163. Label-free Raman microspectroscopy further characterized cellular components of peritoneal macrophages, including nucleic acids, proteins and lipids. Representative Raman images of the false color-coded heat maps are shown in **Figure 1F**. Nuclei-specific peaks in **Figure 1G** showed characteristic peaks at 785 cm^- 1^ (25), 1458 cm ^-1^ (26) and 1655 to 1680 cm^-1^ (25), while protein-specific spectra were identified based on peaks at 1008 cm ^-1^ (27), 1308 cm ^-1^ (27) and 1667 cm^-1^ (28, 29). Characteristic peaks of lipids in Raman spectra are related to the hydrocarbon chain (e.g., 1250 to 1300 cm ^-1^, 1400 to 1500 cm ^-1^) (30). The C-H stretching, which is found in the bands of the higher wavenumber region, is also distinctive of lipid spectra (30). A detailed molecular assignment of the nuclei-, protein- and lipid-specific peaks is summarized in **Table 1**.

**Table 1:** Identified Raman peaks [cm^−1^] and their molecular assignments.

Peaks [cm^−1^]	Assignment	Reference
Nuclei		
785	uracil, thymine, cytosine, O-P-O backbone	(25)
1458	nucleic acid modes	(26)
1655-80	thymine, guanine, cytosine (ring breathing modes)	(25)
Proteins		
1008	phenylalanine	(27)
1308	C-N asymmetric stretching in aromatic amines	(27)
1667	protein bands	(28, 29)
Lipids		
1270	C=C groups (unsaturated fatty acids)	(31)
1440	(CH_2_) (lipids), CH_2_ bending (lipids)	(32, 33)
1655	C=C (lipids; not amide I)	(34)
2844	*v*_s_(=CH_2_)	(30)
3010	unsaturated =CH stretch	(31)

### 3.2 PAL-treated peritoneal macrophages maintain resistance towards oxidative cellular death by upregulating anti-oxidative mechanisms

**Figure 2:**
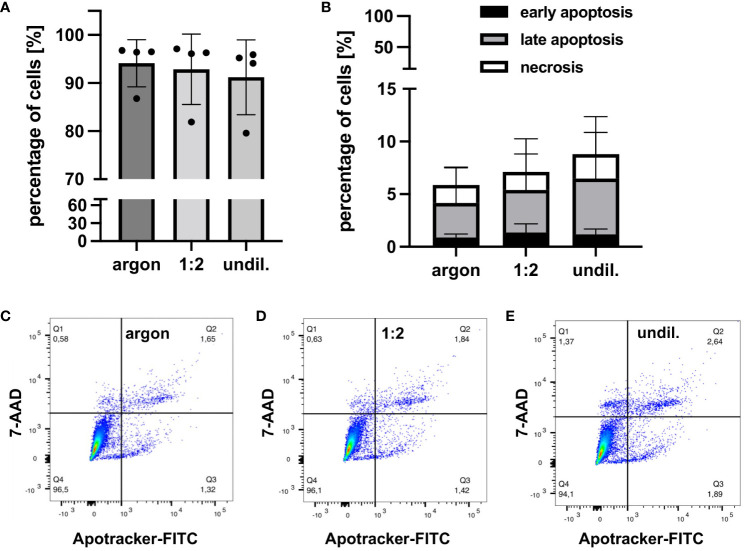
FC analysis of viability, apoptosis and necrosis of PAL-treated peritoneal macrophages. Apotracker-FITC and 7-AAD staining was performed 24 h after PAL treatment of peritoneal macrophages. **(A)** Bar graph shows high viability of the 1:2-diluted and undiluted PAL-treated macrophages. **(B)** Bar graph shows small, non-significant increase in early (black), late (light grey) apoptosis and necrosis (white) of PAL-treated macrophages. **(C–E)** Representative dot plots of one donor for the argon-treated control **(C)**, 1:2-diluted **(D)** and undiluted PAL **(E)**. Shown are mean ± SD, n = 4.

**Figure 3:**
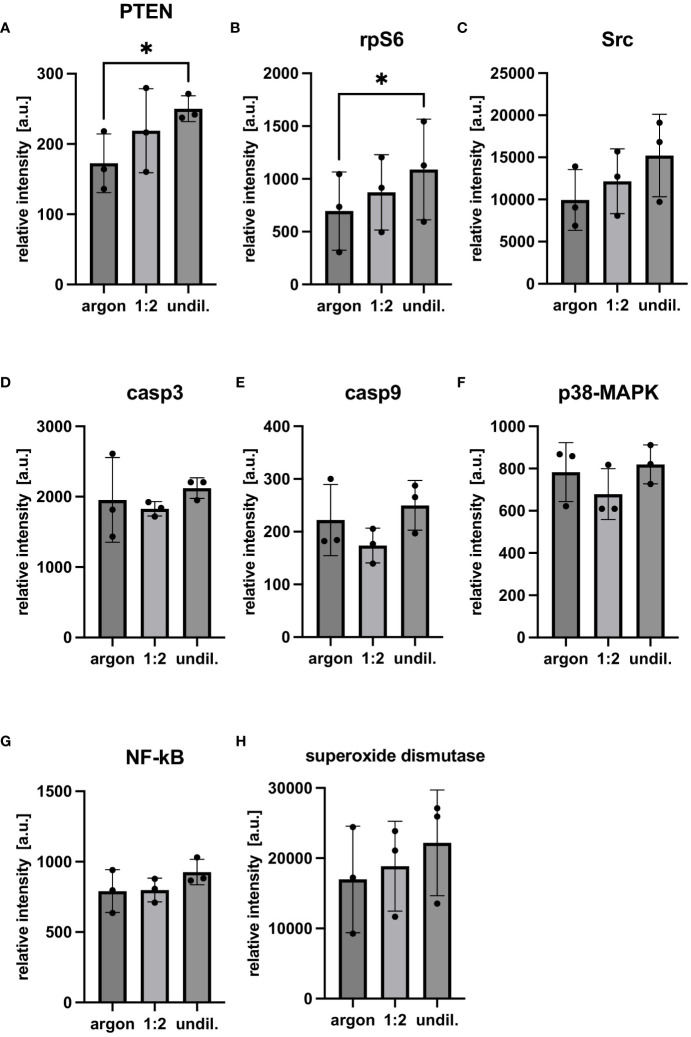
Multiplex protein profiling of PAL-treated peritoneal macrophages. Protein profiling using DigiWest technology was performed with samples frozen 24 h after PAL treatment. Antibody fluorescence intensities of the analytes were normalized to the argon-treated and their respective streptavidin loading control. **(A–C)** show cellular factors related to proliferation, immune response and survival. **(D–G)** show cellular factors and signaling pathways related to apoptosis. **(H)** shows superoxide dismutase, a redox-related enzyme. Statistical comparison was performed with paired Student’s *t*-tests. Shown are mean ± SD, n = 3, * p < 0.05.

Cellular factors related to apoptosis, necrosis and pro-survival pathways were analyzed in PAL-treated macrophages using FC and DigiWest protein profiling. FC staining of PAL-treated macrophages with Apotracker and 7-AAD demonstrated marginal, non-significant levels of apoptosis and necrosis (**Figure 2**). Consistent with the low levels of apoptosis and necrosis, PAL-treated macrophages showed a high viability for the 1:2-diluted and undiluted PAL compared to the argon-treated control (argon-treated control: 94.1 ± 4.9%, 1:2-diluted: 92.9 ± 7.3% and undiluted PAL: 91.2 ± 7.8%). Representative dot plots of one donor for the argon-treated control, 1:2-diluted and undiluted PAL are shown in **Figures 2C–E**. Quadrant 1 (Q1) shows necrotic (Apo-, 7-AAD+), Q2 late apoptotic (Apo+, 7-AAD+), Q3 early apoptotic (Apo+, 7-AAD-) and Q4 viable cells (Apo-, 7-AAD-). Additional apoptosis markers, including the expression of caspases 3 and 9, also showed no significant increase (**Figure 3**). Signal proteins related to immune response control and proliferation, such as proto-oncogene tyrosine-protein kinase (Src, 1:2-diluted: p = 0.0981; undiluted PAL: p = 0.0661), S6 ribosomal protein (rpS6, 1:2-diluted: p = 0.4141; undiluted PAL: p =0.0231) and phosphatase and tensin homolog (PTEN, 1:2-diluted: p = 0.3242; undiluted PAL: p = 0.0306) showed an increased expression. The absence of significant spectral changes at a nuclei level in Raman microspectroscopy further supports the PAL-treated macrophages’ resistance towards oxidative stress-induced cell death (**Supplementary Figure S2**). Superoxide dismutase, a redox-related enzyme, was mildly upregulated for undiluted PAL compared to the argon-treated control (1:2-diluted: p = 0.5827, undiluted: p = 0.1008), which may explain the increased anti-oxidative potential of peritoneal macrophages.

### 3.3 PAL-treated peritoneal macrophages show a moderate pro-inflammatory shift by alteration of their molecular composition and cytokine release

**Figure 4:**
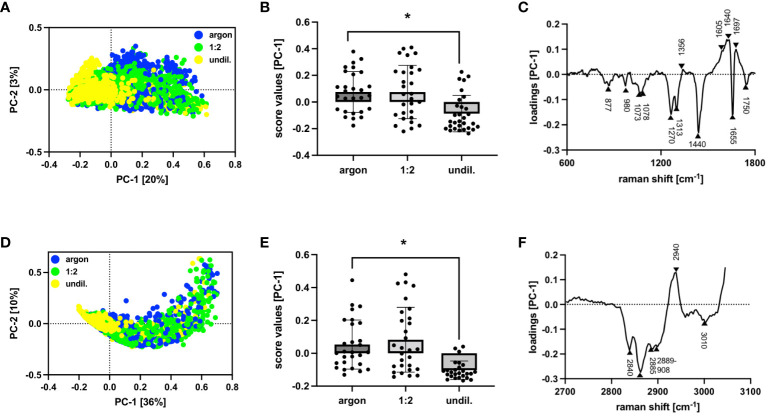
Raman and multivariate analysis of lipid composition in PAL-treated macrophages. Raman and multivariate analysis reveal spectral differences at a lipid level within the fingerprint (600 to 1800 cm^-1^) and higher wavenumber region (2700 to 3100 cm^-1^) in PAL-treated macrophages. **(A)** Score plot of fingerprint region demonstrated separation in PC-1 vs PC-2 of argon-treated control (blue) and undiluted PAL (yellow). **(B)** Average score values of fingerprint region show significant differences of PC-1 for argon-treated control compared to undiluted PAL-treated macrophages. **(C)** Corresponding PC-1 loading plot of fingerprint region indicates changes in lipidome profile for undiluted PAL-treated macrophages. **(D)** Score plot of higher wavenumber region demonstrated separation in PC-1 vs PC-2 of argon-treated control (blue) and undiluted PAL (yellow). **(E)** Average score values of higher wavenumber region show significant differences of PC-1 for argon-treated control compared to undiluted PAL-treated macrophages. **(F)** Corresponding PC-1 loading plot of higher wavenumber region indicates changes in lipidome profile for undiluted PAL-treated macrophages. Shown are statistical comparisons using an unpaired Student’s *t*-test or Mann-Whitney *U* test of average score values ± SD for 28 single cells, n = 3, * p < 0.05.

**Figure 5:**
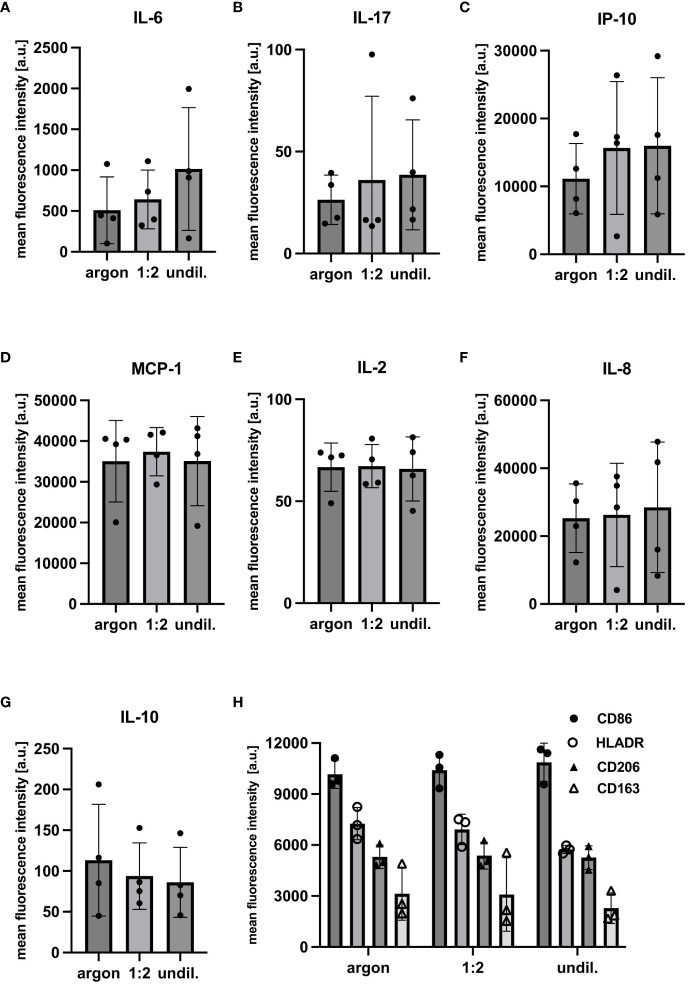
FC analysis of cytokine/chemokine release and surface marker expression of PAL-treated macrophages. FC analysis of cytokine/chemokine release and surface marker expression was performed 24 h after PAL treatment. Mean fluorescence intensities (MFIs) of cytokine/chemokine levels were measured using a bead-based immunoassay. **(A–C)** Tendential increase in pro-inflammatory cytokines and chemokines (IL-6, IL-17 and IP-10). **(D–F)** Other pro-inflammatory cytokines (MCP-1, IL-2 and IL-8) showed no PAL-derived changes. **(G)** Release of anti-inflammatory cytokine, IL-10, showed a moderate decrease. Shown are the mean ± SD, n = 4. **(H)** FC analysis of surface marker expression was performed and surface protein levels are shown as MFIs. HLADR (M1) and CD163 (M2) expression were moderately reduced for the argon-treated control compared to the undiluted PAL-treated macrophages. Shown are mean ± SD, n = 3.

PAL-derived RONS did appear to affect molecular composition, cytokine release and surface marker expression as shown by marker-independent Raman microspectroscopy and FC staining. Two separate PC analyses were performed for the lipidome profile of PAL-treated macrophages, as the higher wavenumber region (2700 to 3100 cm^-1^) concealed spectral differences in the fingerprint region (600 to 1800 cm^-1^). Score plots of the fingerprint and higher wavenumber region in **Figure 4** demonstrated distinct clusters of argon-treated and undiluted PAL-treated macrophages (fingerprint: 1:2-diluted: p = 0.9965, undiluted: p <0.0001; higher wavenumber region: 1:2-diluted: p = 0.9273, undiluted PAL: p <0.0001). Raman peaks at, for example, 1270 cm^-1^ (31), 1440 cm^-1^ (32, 33), 1655 cm^-1^ (34), 2844 cm^-1^ (30) and 3010 cm^-1^ (31) in the loading plots explain spectral differences (**Table 1**). The aforementioned peaks can be assigned to PAL-treated macrophages, indicating the C=C double bond found in unsaturated fatty acids. Further relevant peaks are summarized in **Supplementary Table S1** (35–41). Changes in fatty acid composition and turnover in PAL-treated macrophages may have contributed to a moderate release of pro-inflammatory cytokines. 24 h after PAL treatment seven of the 13 analytes measured, including IL-2, IL-6, IL-8, IL-10, IL-17, IP-10 and MCP-1, were detectable in the cell culture supernatants of the PAL-treated macrophages using a bead-based immunoassay (**Figures 5A–G**). MFIs of the individual analytes measured were averaged (duplicates) and their respective absolute concentrations are summarized in **Supplementary Table S2**. Pro-inflammatory cytokines, including IL-6, IL-17 and IP-10, showed a moderate increase (undiluted PAL: IL-6: p = 0.2837; IL-17: p = 0.4288; IP-10: p = 0.1426). However, chemokine and cytokine release of PAL-treated compared to argon-treated macrophages was not significant due to a high donor-dependent variance. Further pro-inflammatory cytokines, including IL-2, IL-8 and MCP-1, showed no PAL-derived changes. The anti-inflammatory cytokine, IL-10, demonstrated a small decrease, which was higher for undiluted (p = 0.1757) compared to the 1:2-diluted PAL (p = 0.2762). IL-8, IP-10 and MCP-1 were the analytes with the highest absolute concentrations (**Supplementary Table S2**). FC staining of surface marker expression was also performed to analyze changes in polarization (**Figure 5H**). CD86 (M1) and CD206 (M2) showed no changes in MFI. However, the expression of CD163 (M2) (1:2-diluted: p = 0.9049, undiluted PAL: p = 0.1556) and HLA-DR (M1) showed a moderate, non-significant downregulation compared to the argon-treated control (1:2-diluted: p = 0.3346, undiluted PAL: p = 0.0889).

## 4 Discussion

Recent research has shown that plasma-derived oxidative stress is not only limited to selectively killing cancer cells but further modifies the tumor microenvironment, including stromal host and immune cells, and may trigger immunogenic cell death by which dying cancer cells release damage-associated molecular patterns (42–45). A NIPP-modulated immune response may thus restore immunogenicity by boosting adaptive immunity against cancer cells. Bekeschus et al., for example, revealed that NIPP treatment of CT26 colorectal cancer cells was related to a higher expression of immunogenic surface-exposed molecules (e.g., calreticulin) (46). Van Loenhout et al. further showed that as NIPP-treated pancreatic stromal host cells released less immunosuppressive signaling molecules (e.g., TGF-ß), more pro-inflammatory immune cells infiltrated the tumor microenvironment (47). These pro-inflammatory M1-like macrophages are responsible for phagocytosis of cancer cells, antigen presentation and release of cytokines (e.g., IL-6) to recruit natural killer and CD8+ T cells essential for tumor control (17). In addition, Takeda et al. showed that intraperitoneal PAL administration significantly reduced metastatic nodules within mice’s peritoneal cavity without toxic effects (48). Also, ovarian cancer dissemination was suppressed *in vitro* and *in vivo* via lower MMP-9 expression, leading to better long-term survival in a mouse model (49). Compared with intraperitoneal chemotherapy (i.e., HIPEC), which may lead to severe postoperative complications (e.g., sepsis, digestive fistula and adhesive ileus) (49–51), intraperitoneal PAL administration may serve as an adjuvant treatment alternative for peritoneal metastasis with fewer adverse events and minimal cytotoxicity to healthy tissue (4, 48). This study thus aimed to identify PAL-derived molecular and immunomodulatory effects on mature human tissue-resident peritoneal macrophages. While cellular effects due to long-lived nitrates (NO_3_-), nitrites (NO_2_-), and hydrogen peroxide (H_2_O_2_) formed by plasma-liquid interactions are shown (45), other effects due to direct treatment (e.g., short-lived species, UV radiation, electromagnetic fields) could not be observed (1).

FC characterization revealed co-expression of M1 and M2 surface markers of the isolated GATA6+ macrophages. Co-stimulatory molecules, CD86 and HLADR, responsible for antigen presentation and T cell activation, are frequently identified with M1 macrophages (13, 17). Higher expression of scavenger receptors CD163 and mannose receptors CD206 indicate an M2-like phenotype (13, 16, 17). Tumor-associated macrophages strongly express CD163, and the density of these macrophages negatively influences gastric cancer growth and metastasis (52). FC characterization of the isolated macrophages showed higher expression of M1 surface markers compared to M2 surface markers. Expression of the surface markers HLADR and CD163 was moderately reduced in PAL-treated macrophages, whereas CD86 and CD206 did not differ notably from the argon-treated control. Possibly, no distinct phenotype shift was observed because of the maturity of the tissue-resident macrophages. Wang et al. demonstrated the different biological characteristics of murine macrophages derived from the peritoneal cavity, spleen and bone marrow, indicating that peritoneal macrophages with high levels of MHC II and CD86 surface marker expression were the most mature and showed lower proliferative potential (53). Alternatively, damage to the cellular membrane via PAL-derived lipid peroxidation may explain reduced surface marker expression. Superoxide radicals (O_2_^•−^) can interfere with hydrogen peroxide (H_2_O_2_) and nitric oxide (NO) to trigger lipid peroxidation, leading to altered cellular membrane permeability and fluidity (54, 55).

PAL-treated macrophages further showed a high resistance towards PAL-derived oxidative stress and cellular death. Although PAL-derived RONS may alter cell membrane integrity and promote apoptosis in cancer cells (56–58), the majority of PAL-treated macrophages maintained high levels of viability and minimal, non-significant levels of apoptosis in FC Apotracker/7-AAD co-staining. Apotracker identifies externalized phosphatidylserine residues in apoptosis (59), whereas viable cells with intact cellular membranes are impermeable to 7-AAD (60). Equipped with increased GSH redox signaling, higher levels of DNA repair proteins and ROS reductase, macrophages have been described to be less sensitive towards higher intracellular ROS levels, which are also present in oxidative burst during phagocytosis (61, 62). As such, NIPP-treated macrophages were demonstrated to be less susceptible to oxidative stress compared to other PBMC-derived leukocyte populations (63). Protein profiling also revealed that PAL-treated macrophages mildly increased their expression of the anti-oxidant enzyme superoxide dismutase, which can catalyze the dismutation of the superoxide radical (O_2_^•−^) to hydrogen peroxide (H_2_O_2_) and molecular oxygen (O_2_) (64). Hwang et al. showed that superoxide dismutase supplementation attenuated uncontrolled inflammatory response and apoptosis via blocking of p38-MAPK/NF-κB pathways (64). Upregulation of superoxide dismutase may also reduce apoptosis by decreasing mitochondrial release of cytochrome c (65). Further apoptosis markers and pathways (e.g., casp3, casp9 and p38-MAPK) demonstrated no significant upregulation in protein profiling of PAL-treated macrophages. Rather cell signaling and regulation pathways relevant for immune response and proliferation showed significant upregulation. PTEN, for example, promotes inflammatory responses via the release of pro-inflammatory cytokines (e.g., IL-6) (66). Src kinase, also relevant for immune response control of macrophages, is involved with their functional activation (67).

Multivariate analysis of spectral data allowed for the biomolecular characterization of cellular structures, including nuclei, proteins and lipids, of PAL-treated macrophages. The potential of Raman imaging to determine PAL-derived changes has already been analyzed in cervical tissue, peritoneal fibroblasts and mesothelial cells (6, 68). Proteins and lipids were previously identified as cellular structures most reactive to demonstrate macrophage activation in Raman imaging (69). Analysis of their lipidome profile revealed that Raman peaks at 1270 cm^-1^ (31), 1440 cm^-1^ (32, 33), 1655 cm^-1^ (34) and 3010 cm^-1^ (31) may explain the clustering behavior of the argon-treated control and PAL-treated macrophages in the score plots. These aforementioned peaks can be assigned to (undiluted) PAL-treated macrophages and describe the C=C double bond of unsaturated fatty acids, thereby indicating an altered degree of saturation in fatty acid composition. Montenegro-Burke et al. demonstrated that macrophage phenotypes have different fatty acid compositions (70). M1 macrophages are characterized by higher intensities of cholesterol esters, diacylglycerols and triglycerides, including a higher proportion of unsaturated triglycerides, especially polyunsaturated fatty acids (71). Cholesterol and triglyceride ester-containing lipid droplets are relevant for inflammatory response and may be utilized as a substrate pool for pro-inflammatory cytokines (e.g., IL-1ß, IL-6) (71, 72). Changes in lipids and their metabolites may affect macrophages’ polarization and response to pathogens, phagocytosis and inflammation (73). Distinguishing in-depth between structural and molecular, as well as transient and permanent damage of cell membranes, requires further studies (i.e., mass spectrometry, electron microscopy, protein profiling) to reveal structural damage and up-/downregulation of lipid-metabolism-related factors due to PAL treatment. Nonetheless, changes in the lipidome profile of PAL-treated macrophages were consistent with observations of a tendential increase of pro-inflammatory cytokines/chemokines (IP-10, IL-6 and IL-17) and a decrease of the anti-inflammatory cytokine IL-10 in the bead-based immunoassay. However, these PAL-derived changes in the cytokine/chemokine release were not significant due to the high donor-dependent variance of primary isolated human tissue-resident peritoneal macrophages. IP-10 (CXCL10), for example, demonstrated immunomodulatory potential to recruit APCs in glioma and melanoma murine tumor models (74). However, IP-10 may also partake in tumor expansion if the receptor CXCR3 is overexpressed in cancer cells. The aforementioned PAL-derived changes in cytokine/chemokine release align with other findings (75, 76). Cheng et al., for example, showed a higher release of IL-2 and IL-6 and a lower IL-10 release in NIPP-treated peritoneal elicited murine macrophages (75).

Our findings suggest that human tissue-resident peritoneal macrophages are extremely resistant towards PAL-derived oxidative stress via upregulated pro-survival and anti-oxidative pathways. NIPP may modulate a moderate pro-inflammatory response by modifying their lipid composition and cytokine release, thereby complementing the aforementioned anti-tumoral activity of NIPP. However, the present study is limited to a 2D cell culture model. 3D cell models (e.g., organoids, spheroids, or tumor-on-a-chip) or murine models better represent the *in vivo* environment and are more predictive of PAL-derived immunomodulatory effects on solid tumors. These must validate the 2D cell culture *in vitro* findings under more *in vivo* (-like) conditions.

## Data availability statement

The raw data supporting the conclusions of this article will be made available by the authors, without undue reservation.

## Ethics statement

The studies involving humans were approved by Institutional Ethics Committee of the Medical Faculty of the Eberhard Karls University Tübingen (protocol codes 649-2017BO2, approval: 12 January 2018 and 495/2018BO2, approval: 19 October 2018). The studies were conducted in accordance with the local legislation and institutional requirements. The participants provided their written informed consent to participate in this study.

## Author contributions

LS-R: Conceptualization, Data curation, Formal analysis, Investigation, Methodology, Writing – original draft. JM: Data curation, Formal analysis, Methodology, Writing – review & editing. DC: Data curation, Formal analysis, Methodology, Writing – review & editing. MH: Data curation, Formal analysis, Investigation, Methodology, Writing – review & editing. FS-R: Methodology, Supervision, Writing – review & editing. TB: Data curation, Investigation, Methodology, Writing – review & editing. JA: Resources, Writing – review & editing. CB: Resources, Writing – review & editing. MT: Methodology, Resources, Supervision, Writing – review & editing, Conceptualization. SB: Funding acquisition, Resources, Writing – review & editing, Conceptualization, Supervision. KS-L: Funding acquisition, Resources, Writing – review & editing, Conceptualization, Supervision. MW: Conceptualization, Formal analysis, Funding acquisition, Methodology, Resources, Supervision, Validation, Writing – review & editing, Investigation, Project administration.
