# Supplementary material for: Human tissue-resident peritoneal macrophages reveal resistance towards oxidative cell stress induced by non-invasive physical plasma
Source: Front Immunol. 2024 Mar 5;15:1357340. doi: 10.3389/fimmu.2024.1357340 (PMC10949891; doi:10.3389/fimmu.2024.1357340)
Supplement: Supplementary file 1 [file DataSheet_1.docx]

Supplementary Material

# Supplementary Figures and Tables

## Supplementary Figures

**Supplementary Figure 1.** **Gating strategy for FC surface marker expression of PAL-treated macrophages.** Representative FC dot plots show gating strategy, which included the removal of cell debris (FSC vs SSC, A), doublets (FSC-A vs SSC-A, B) and dead cells (FSC vs 7-AAD, C) to determine MFIs. Unstained control visualized background autofluorescence to identify negative cell populations and set gates for cells stained with CD86-PE (D), HLADR- FITC (E), CD163-PE-Cy7 (F) and CD206-BV421 (G).

**Supplementary Figure 2.** **Raman and multivariate analysis of nuclei in PAL-treated macrophages.** Raman and multivariate analysis reveal no spectral differences at a nuclei level within the fingerprint region in PAL-treated macrophages. (A) Score plot of fingerprint region demonstrated no separation in PC-2 vs PC-3 of argon- (blue), 1:2-diluted (green) and undiluted (yellow) PAL-treated macrophages, (B) as indicated in statistical comparisons of the average score values. Shown are the average score values ± SD for $\geq$28 single cells, n = 3.

## Supplementary Tables

**Supplementary Table 1. Raman shifts and the respective molecular assignments.**

| **Peaks [cm^−1^]** | **Assignment** | **Reference** |
| --- | --- | --- |
| 877 | C-C-N^+^ symmetric stretching (lipids) | (35) |
| 980 | =CH bending (lipids) | (35) |
| 1073 | triglycerides (fatty acids) | (36) |
| 1078 | C-C or C-O stretching mode (phospholipids) | (37) |
| 1313 | CH_3_CH_2_ twisting mode (lipids, collagen) | (38, 39) |
| 1638 | intermolecular bending mode of water | (39) |
| 1697 | amide I | (32) |
| 1750 | C=O (lipids), n(C=C) (lipids, fatty acids) | (34, 40) |
| 2853 - 81 | CH_2_ symmetric stretch (lipids), CH_2_ asymmetric stretch (lipids, proteins) | (33) |
| 2889-908 | CH_2_ asymmetric stretch (lipids) | (33) |
| 2940 | C-H vibrations (lipids, proteins), $\nu$_as_CH_2_, (lipids, fatty acids) | (40, 41) |

**Supplementary Table 2. Average MFIs and absolute concentrations of cytokine/chemokine release in PAL-treated macrophages.**

|  | | **average MFI [a.u.]** | | | | **average absolute concentration [pg / mL]** | | | |
| --- | --- | --- | --- | --- | --- | --- | --- | --- | --- |
| **donor**  **(n = 4)** | | 1 | 2 | 3 | 4 | 1 | 2 | 3 | 4 |
| **IL-6** | argon | 446 | 102 | 412 | 1075 | 122 | 24.7 | 112 | 281 |
|  | 1:2 | 1110 | 324 | 397 | 736 | 289 | 88.6 | 108 | 197 |
|  | undil. | 989 | 165 | 1995 | 909 | 260 | 43.5 | 501 | 240 |
| **IL-17** | argon | 40 | 15 | 34 | 18 | 6.67 | 1.96 | 5.63 | 2.55 |
|  | 1:2 | 16 | 16 | 98 | 13 | 2.33 | 2.33 | 15.2 | 1.69 |
|  | undil. | 22 | 40 | 76 | 17 | 3.41 | 6.73 | 12.3 | 2.37 |
| **IP-10** | argon | 12625 | 8152 | 17705 | 6054 | 565 | 364 | 858 | 277 |
|  | 1:2 | 17276 | 16376 | 26368 | 2659 | 830 | 773 | 1618 | 148 |
|  | undil. | 17597 | 11249 | 29187 | 5892 | 850 | 499 | 1980 | 271 |
| **IL-2** | argon | 72 | 49 | 74 | 72 | 5.63 | 2.73 | 5.85 | 5.74 |
|  | 1:2 | 81 | 58 | 71 | 59 | 6.73 | 3.99 | 5.48 | 4.08 |
|  | undil. | 74 | 45 | 81 | 63 | 5.94 | 2.22 | 6.81 | 4.52 |
| **IL-8** | argon | 30348 | 12278 | 35577 | 22954 | 1847 | 372 | 2899 | 1017 |
|  | 1:2 | 34854 | 28463 | 37569 | 4120 | 2631 | 1594 | 3254 | 102 |
|  | undil. | 47838 | 16048 | 41765 | 8341 | 7633 | 548 | 4549 | 223 |
| **IL-10** | argon | 116 | 45 | 85 | 206 | 9.39 | 2.65 | 6.50 | 17.2 |
|  | 1:2 | 86 | 61 | 75 | 153 | 6.64 | 4.16 | 5.58 | 12.6 |
|  | undil. | 70 | 46 | 83 | 146 | 5.08 | 2.70 | 6.28 | 12.1 |
| **MCP-1** | argon | 40561 | 20068 | 39247 | 40337 | >10000 | 1670 | >10000 | >10000 |
|  | 1:2 | 41575 | 29348 | 42102 | 36578 | >10000 | 3809 | >10000 | 7917 |
|  | undil. | 41213 | 19177 | 43148 | 36858 | >10000 | 1523 | >10000 | 8186 |
